# Supplementary material for: Comparative genomics provides new insights into the diversity, physiology, and sexuality of the only industrially exploited tremellomycete: Phaffia rhodozyma
Source: BMC Genomics. 2016 Nov 9;17:901. doi: 10.1186/s12864-016-3244-7 (PMC5103461; doi:10.1186/s12864-016-3244-7)
Supplement: Additional file 6: — List of orphan genes with links to PFAM (related to Additional file 1: Table S1). (ZIP 1428 kb) [file 12864_2016_3244_MOESM6_ESM.zip › PFAM_UNIQUE/G01150_P.html]

#                                                                            --- full sequence --- -------------- this domain -------------   hmm coord   ali coord   env coord
  
# target name        accession   tlen query name           accession   qlen   E-value  score  bias   #  of  c-Evalue  i-Evalue  score  bias  from    to  from    to  from    to  acc description of target
  
#------------------- ---------- ----- -------------------- ---------- ----- --------- ------ ----- --- --- --------- --------- ------ ----- ----- ----- ----- ----- ----- ----- ---- ---------------------
  
G01150\_P             -            282 7tm\_1                PF00001.16   257     0.014   13.2   0.0   1   1   2.2e-05     0.019   12.8   0.0   147   199    82   133    49   171 0.79 - 7 transmembrane receptor (rhodopsin family)  
G01150\_P             -            282 ABC\_tran\_2           PF12848.2     85      0.66    8.5   7.6   1   1    0.0013       1.1    7.8   7.6    15    63   102   150   101   179 0.72 - ABC transporter  
G01150\_P             -            282 Caldesmon            PF02029.10   492       3.9    4.4  10.2   1   1     0.054       5.7    3.9  10.2   307   362   107   163   102   184 0.81 - Caldesmon  
G01150\_P             -            282 Daxx                 PF03344.10   713         5    4.1  13.4   1   1     0.084       6.9    3.7  13.4   469   628   115   268   105   279 0.56 - Daxx Family  
G01150\_P             -            282 DUF1183              PF06682.7    319     0.011   14.1   2.6   1   1   1.7e-05     0.025   12.8   2.6   157   239    86   172    57   265 0.70 - Protein of unknown function (DUF1183)  
G01150\_P             -            282 DUF2681              PF10883.3     87    0.0011   17.8   0.0   1   1   9.5e-07    0.0019   17.1   0.0     8    59    88   140    83   154 0.80 - Protein of unknown function (DUF2681)  
G01150\_P             -            282 DUF3609              PF12259.3    361     0.032   11.6   0.0   1   1   7.6e-06     0.046   11.0   0.0   305   350    88   132    66   136 0.68 - Protein of unknown function (DUF3609)  
G01150\_P             -            282 DUF4638              PF15472.1    268     0.057   11.7   1.6   1   1   1.4e-05     0.082   11.1   1.6    32   102    95   166    92   227 0.61 - Domain of unknown function (DUF4638)  
G01150\_P             -            282 FxsA                 PF04186.8    120     0.052   11.8   0.0   1   1   1.5e-05     0.089   11.1   0.0    22    65    83   126    81   138 0.88 - FxsA cytoplasmic membrane protein  
G01150\_P             -            282 MIP-T3               PF10243.4    539       8.9    3.3  18.6   1   1      0.08        10    3.1  18.6   132   208   110   184   104   273 0.48 - Microtubule-binding protein MIP-T3  
G01150\_P             -            282 NARP1                PF12569.3    517       5.7    4.2   7.7   1   1     0.029       7.8    3.8   7.7   416   466   107   157   103   171 0.76 - NMDA receptor-regulated protein 1  
G01150\_P             -            282 PGA2                 PF07543.7    140      0.63    8.5   3.7   1   1   0.00099       1.2    7.6   3.7    23    76    96   147    76   183 0.69 - Protein trafficking PGA2  
G01150\_P             -            282 PLRV\_ORF5            PF01690.12   465      0.45    8.2   5.5   1   1    0.0018      0.52    8.0   5.5   280   438   117   264    62   279 0.40 - Potato leaf roll virus readthrough protein  
G01150\_P             -            282 UPF0542              PF15086.1     74    0.0065   14.9   0.0   1   1   5.3e-06     0.016   13.6   0.0    17    67    72   126    69   147 0.71 - Uncharacterised protein family UPF0542  
G01150\_P             -            282 Utp14                PF04615.8    735       8.1    3.1   8.5   1   1      0.12        11    2.7   8.5   382   450   116   184   102   276 0.43 - Utp14 protein
